# Platelet factor 4 induces bone loss by inhibiting the integrin α5‐FAK‐ERK pathway

**DOI:** 10.1002/ame2.12342

**Published:** 2023-08-11

**Authors:** Wei Li, Qiwei Zhang, Ranli Gu, Lijun Zeng, Hao Liu

**Affiliations:** ^1^ Department of Oral Pathology, Peking University School and Hospital of Stomatology, National Center for Stomatology, National Clinical Research Center for Oral Diseases, National Engineering Research Center of Oral Biomaterials and Digital Medical Devices, Beijing Key Laboratory of Digital Stomatology, National Health Commission Key Laboratory of Digital Technology of Stomatology Peking University Beijing China; ^2^ Research Unit of Precision Pathologic Diagnosis in Tumors of the Oral and Maxillofacial Regions Chinese Academy of Medical Sciences Beijing China; ^3^ Department of Orthopedics, Beijing Hospital and National Center of Gerontology and Institute of Geriatrics Medicine Chinese Academy of Medical Sciences Beijing China; ^4^ Department of Orthopedics Beijing Eden Hospital Beijing China; ^5^ Department of Prosthodontics, Peking University School and Hospital of Stomatology, National Center for Stomatology, National Clinical Research Center for Oral Diseases, National Engineering Research Center of Oral Biomaterials and Digital Medical Devices, Beijing Key Laboratory of Digital Stomatology, National Health Commission Key Laboratory of Digital Technology of Stomatology Peking University Beijing China; ^6^ The Central Laboratory, Peking University School and Hospital of Stomatology, National Center for Stomatology, National Clinical Research Center for Oral Diseases, National Engineering Research Center of Oral Biomaterials and Digital Medical Devices, Beijing Key Laboratory of Digital Stomatology, National Health Commission Key Laboratory of Digital Technology of Stomatology Peking University Beijing China

**Keywords:** bone loss, bone marrow mesenchymal stem cells, integrin α5, osteogenesis, platelet factor 4

## Abstract

**Background:**

The effect of platelet factor 4 (PF4) on bone marrow mesenchymal stem cells (BMMSCs) and osteoporosis is poorly understood. Therefore, this study aimed to evaluate the effects of PF4‐triggered bone destruction in mice and determine the underlying mechanism.

**Methods:**

First, in vitro cell proliferation and cell cycle of BMMSCs were assessed using a CCK8 assay and flow cytometry, respectively. Osteogenic differentiation was confirmed using staining and quantification of alkaline phosphatase and Alizarin Red S. Next, an osteoporotic mouse model was established by performing bilateral ovariectomy (OVX). Furthermore, the PF4 concentrations were obtained using enzyme‐linked immunosorbent assay. The bone microarchitecture of the femur was evaluated using microCT and histological analyses. Finally, the key regulators of osteogenesis and pathways were investigated using quantitative real‐time polymerase chain reaction and Western blotting.

**Results:**

Human PF4 widely and moderately decreased the cell proliferation and osteogenic differentiation ability of BMMSCs. Furthermore, the levels of PF4 in the serum and bone marrow were generally increased, whereas bone microarchitecture deteriorated due to OVX. Moreover, in vivo mouse PF4 supplementation triggered bone deterioration of the femur. In addition, several key regulators of osteogenesis were downregulated, and the integrin α5‐focal adhesion kinase‐extracellular signal‐regulated kinase (ITGA5‐FAK‐ERK) pathway was inhibited due to PF4 supplementation.

**Conclusions:**

PF4 may be attributed to OVX‐induced bone loss triggered by the suppression of bone formation in vivo and alleviate BMMSC osteogenic differentiation by inhibiting the ITGA5‐FAK‐ERK pathway.

## Graphical abstract

Platelet factor 4 alleviated proliferation and osteogenic differentiation of bone marrow mesenchymal stem cell s through inhibiting the integrin α5‐focal adhesion kinase‐extracellular signal‐regulated kinase pathway, which may be related to ovariectomy‐induced bone loss.

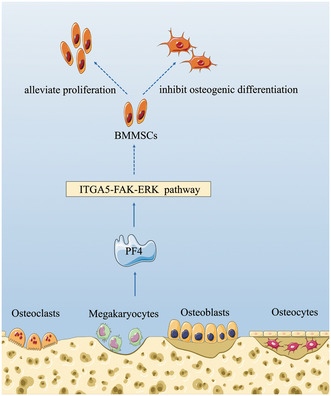

## 1 INTRODUCTION

Osteoporosis is a chronic syndrome characterized by the loss of bone mass and destruction of the bone microarchitecture, which finally leads to excessive skeletal fragility.^1^ Mechanistically, osteoporosis occurs due to an imbalance between osteoblast‐mediated bone formation and bone resorption by osteoclasts (i.e., a higher level of bone resorption than bone formation).^2^ Current strategies for osteoporotic prevention and treatment focus on promoting bone regeneration, suppressing bone resorption, or a combination of both.^3^ Recently, some targeted drugs for osteoporosis have been approved for clinical use and exhibit excellent curative effects.^4^, ^5^, ^6^

As a predominant source of osteoblasts, it is crucial to maintain osteogenic differentiation and proliferation of bone marrow mesenchymal stem cells (BMMSCs) to preserve bone homeostasis.^7^ Furthermore, several studies have revealed that BMMSC‐mediated osteogenic differentiation and proliferation can be downregulated or impaired by multiple factors in the bone marrow (BM), which is markedly associated with osteoporosis.^8^, ^9^, ^10^

It has been well established that the osteogenic differentiation and proliferation of BMMSCs is suppressed by several cytokines^11^, ^12^, ^13^; however, the relationship between some cytokines and BMMSCs is unclear. Platelet factor 4 (PF4), also termed C‐X‐C motif ligand 4 (CXCL4), is a 7.8‐kDa platelet α‐granule protein synthesized by megakaryocytes in the BM and belongs to the CXC chemokine subfamily.^14^ In general, PF4 plays multiple roles in modulating hematopoiesis, angiogenesis, and cell proliferation.^15^, ^16^, ^17^, ^18^, ^19^, ^20^ Recent research has demonstrated that PF4 secretion by megakaryocytes can directly regulate hemopoietic stem cell (HSC) quiescence in the local HSC niche of the BM. Similarly, the percentage of HSCs was lower after treatment with PF4 compared with those treated with PBS.^21^ However, the specific role of PF4 in BMMSCs has never been elucidated. Given the multiple physiological effects of PF4, we speculated that it may downregulate the proliferation or osteogenic differentiation of BMMSCs, which may be relevant to osteoporosis.

Recently, several studies have elucidated that platelet factors regulate the function of recipient cells through integrins, which belong to the family of transmembrane adhesion receptors formed by the 19 α and 8 β subunits.^22^, ^23^, ^24^ These subunits can interact to form 24 heterodimeric receptors via noncovalent binding, which exist on the recipient cell surface to recognize and respond to various proteins, such as PF4, platelet factor XIII, or osteopontin.^25^ Therefore, integrins play roles in regulating cellular proliferation, migration, and differentiation.^26^ Moreover, integrin signaling can be transduced into the cytoplasm by intracellular signaling molecules, such as focal adhesion kinase (FAK), which can directly bind to focal adhesion sites located in the cytoplasmic domain of integrin. In addition, several downstream molecules are involved in integrin‐FAK pathways, such as extracellular signal‐regulated kinase (ERK), which can directly inhibit caspase 9 activation by phosphorylation of Thr^125^ located in the conserved mitogen‐activated protein kinase consensus site.^27^

Therefore, this study aimed to confirm the relationship between PF4 and osteoporosis, evaluate the effects of PF4‐triggered bone destruction in mice in vivo, and determine the mechanism underlying the suppression of osteogenesis or proliferation of BMMSCs induced by PF4.

## 2 MATERIALS AND METHODS

### 2.1 Cell proliferation and migration assays

To explore the effects of PF4 on BMMSC proliferation and migration, CCK8 and transwell assays were performed.^28^, ^29^ Briefly, human BMMSCs (Science Cell Research Laboratories) were cultivated in maintenance medium (Dulbecco's Modified Eagle's Medium containing 10% fetal bovine serum, 100 U/mL of penicillin G, and 100 mg/mL of streptomycin) (GIBCO) at 37°C in an atmosphere of 5% CO_2_. BMMSCs at passages 3–5 were cocultured with PBS, 0.2 μg/mL of recombinant human PF4 (hPF4, Sigma‐Aldrich), 1 μg/mL of hPF4, and 1 μg/mL of hPF4 and 30 μg/mL of heparin (hPF4 antagonist) (Sigma‐Aldrich). CCK8 (Dojindo Laboratories) and transwell assays were performed according to the manufacturer's instructions. All cell‐based experiments were repeated at least thrice.

### 2.2 Flow cytometry

To investigate the effects of PF4 on the cell cycle, BMMSCs treated as mentioned earlier were measured using flow cytometry (FCM).^30^ Briefly, BMMSCs were harvested, resuspended in PBS, and fixed using cold alcohol. Subsequently, PI/RNase staining buffer biosciences, Inc. (BD) was added to the BMMSCs and incubated. Finally, the cell cycle was tested using a BD LSRII flow cytometer BD.

### 2.3 Staining and quantification of alkaline phosphatase and Alizarin Red S

To investigate the osteogenic effect of PF4 on BMMSCs, staining and quantification of alkaline phosphatase (ALP) and Alizarin Red S (ARS) were undertaken.^31^ First, hBMMSCs were seeded at 40 000 cells/well in six‐well plates and cultured in an osteogenic medium (OM, maintenance medium containing 10 nM dexamethasone, 10 mM β‐glycerophosphate, and 0.2 mM l‐ascorbic acid [Sigma‐Aldrich]). hBMMSCs were first stained with an NBT/BCIP staining kit (CoWin Biotech) after culturing for 1 week for ALP and with a 2% ARS staining solution (Sigma‐Aldrich) after culturing for 3 weeks for ARS.

### 2.4 Animals and administration procedure

Eight‐week‐old female C57BL6/J mice (Vital River Inc.) were housed in the specific‐pathogen‐free room in accordance with the Guide for the Care and Use of Laboratory Animals. All animal experiments were approved by the Animal Care and Use Committee of Peking University Health Science Center (approval number: LA2019019; Beijing, China). First, to explore the relationship between PF4 and osteoporosis, a classic osteoporotic animal model was established via a ligaturing bilateral OVX. Generally, 60 mice were split into 10 groups of 6 mice, and the bilateral OVX was ligated using standard methods.^32^ After surgery, euthanasia was performed on each group every 2 weeks (at least) until week 12. The serum and BM of the tibia were obtained to detect the concentration of PF4 using enzyme‐linked immunosorbent assay (ELISA) or the relative expression of *Pf4* using quantitative real‐time polymerase chain reaction (qPCR). To obtain BM from the tibia, the tibia was dissected free from soft tissue thoroughly, and the tip of the tibia was removed using a rongeur. Next, the BM was flushed with maintenance medium (for ELISA) or TRIzol (for qPCR) (Life Technologies) by inserting a sterilized syringe needle into one end of the bone. In addition, the femurs were dissected for microCT and histology. To investigate the effects on PF4‐induced bone loss, 30 mice were split into three groups comprising 10 mice and treated with PBS, 2 μg of recombinant mouse PF4 (mPF4), and 5 μg of mPF4 (i.p., dissolved in normal saline, every other day) (Sigma‐Aldrich). The mice were euthanized 8 weeks after the injection. Tissues were obtained using the aforementioned methods.

### 2.5 MicroCT analysis

To evaluate the differences in bone morphometry in these groups, microCT was performed using an Inveon MM system (Siemens).^33^ Briefly, the images of the femurs were obtained using the following parameters: pixel size, 9.21 μm; current, 220 μA; voltage, 60 kV; and exposure time, 1500 ms. The bone mineral density (BMD) and bone morphometric parameters were analyzed using Inveon Research Workplace software (Siemens).

### 2.6 Histological analysis

To further confirm PF4‐induced bone loss, the femurs were decalcified using a 10% ethylenediaminetetraacetic acid solution after fixation in formalin. Five‐micron slices were obtained using standard procedures and stained with H&E.

To investigate the histomorphometric changes in bone dynamics, undecalcified bone slicing and subsequent analyses were performed.^34^ Briefly, on the 10th and 3rd days prior to euthanasia, the mice were intraperitoneally injected with alizarin‐3‐methyliminodiacetic acid (30 mg/kg, Sigma‐Aldrich) and calcein (10 mg/kg, Sigma‐Aldrich), respectively. The femurs were ground and polished into ~50 μm slices using the EXAKT precision cutting and grinding system (EXAKT Apparatebau). Subsequently, dynamic histomorphometric parameters were analyzed using Bioquant software (BioQuant).

### 2.7 Enzyme‐linked immunosorbent assay

An ELISA was used to test the concentration of PF4 in the serum and BM from the tibia. In brief, the serum was separated from the blood, and BM supernatants were flushed out using PBS. A PF4 ELISA kit (R&D Systems) was used to detect the concentration of PF4 in the serum and BM according to the manufacturer's standard operating procedures. At a minimum, the samples were tested in duplicate.

### 2.8 RNA extraction and qPCR

The total RNA of BMMSCs treated with different factors was extracted using an RNeasy Mini Kit (Qiagen). cDNA was synthesized using a PrimeScript RT Reagent Kit (Takara). qPCR was performed using a 7500 real‐time PCR detection system (Applied Biosystems). The primers are provided in Table S1, and the procedure was performed as follows: 95°C for 10 min, followed by 40 cycles of 95°C for 15 s and 60°C for 1 min. Finally, the data were analyzed using the 2^−ΔCT^ method through *GAPDH/Gapdh* for normalization.^35^

### 2.9 Construction of ITGA5i and ITGA5 shRNA lentivirus

The silencing/overexpression of ITGA5 was performed using an ITGA5‐targeting (ITGA5i) oligonucleotide sequence or an ITGA5 overexpression lentivirus designed by GeneChem (Shanghai, China), as previously described.^36^ Briefly, the ITGA5i oligonucleotide sequence was CTCCTATATGTGACCAGAGTT, and the ITGA5 overexpression lentivirus contained only the full transcript of the *ITGA5* gene. In addition, the lentiviral work vector was Ubi‐MCS‐3FLAG‐SV40‐EGFP‐IRES‐puromycin. The BMMSCs were infected with the abovementioned lentivirus at a multiplicity of infection of 20.

### 2.10 Western blotting

Western blotting (WB) was performed using the standard procedure as previously described.^37^ Briefly, the cells were lysed to obtain total protein using radioimmunoprecipitation assay buffer mixed with protease inhibitor (Solarbio). Then, the lysates were centrifuged at 10 000*g* for 30 min at 4°C. The total protein concentrations were detected using a Pierce BCA protein assay kit (Thermo Scientific). Protein extracts (20 μg) were separated using 10% sodium dodecyl sulfate–polyacrylamide gel electrophoresis and transferred onto polyvinylidene difluoride membranes (Merck Millipore). In addition, the membranes were incubated at 4°C overnight with respective primary antibodies (Abcam), including CCND1 (ab40754, 1:2000), CDK4 (ab108357, 1:1000), CDKN1B (ab32034, 1:5000), SP7 (ab209484, 1:1000), SPP1 (ab214050, 1:1000), SPARC (ab207743, 1:1000), RUNX2 (ab236639, 1:1000), COL1A1 (ab138492, 1:1000), ITGA2 (ab181548, 1:5000), ITGA5 (ab150361, 1:10000), ITGA6 (ab181551, 1:2000), ITGB1 (ab52971, INTEGRIN BETA 1, 1:10000), ITGB3 (ab119992, 1:1000), ITGB4 (ab133682, 1:1000), FAK (ab40794, 1:2000), p‐FAK (ab81298, 1:1000), ERK 1/2 (ab184699, 1:10000), p‐ERK 1/2 (ab201015, 1:1000), and GAPDH (ab181602, 1:10000), and then detected on immunoreactive protein bands after incubation for 1 h using an ECL kit (CWBIO) with horseradish peroxidase‐linked anti‐rabbit secondary antibodies.

### 2.11 Statistical analysis

Statistical analysis was conducted using IBM SPSS Statistics 20.0 software (SPSS). An independent two‐tailed Student's *t‐*test or one‐way analysis of variance with a post hoc analysis using the least significant difference was applied. Data are expressed as mean ± standard deviation (SD). A threshold *p*‐value < 0.05 was considered statistically significant.

## 3 RESULTS

### 3.1 PF4 decreases the cell proliferation and migration ability of BMMSCs

**FIGURE 1:**
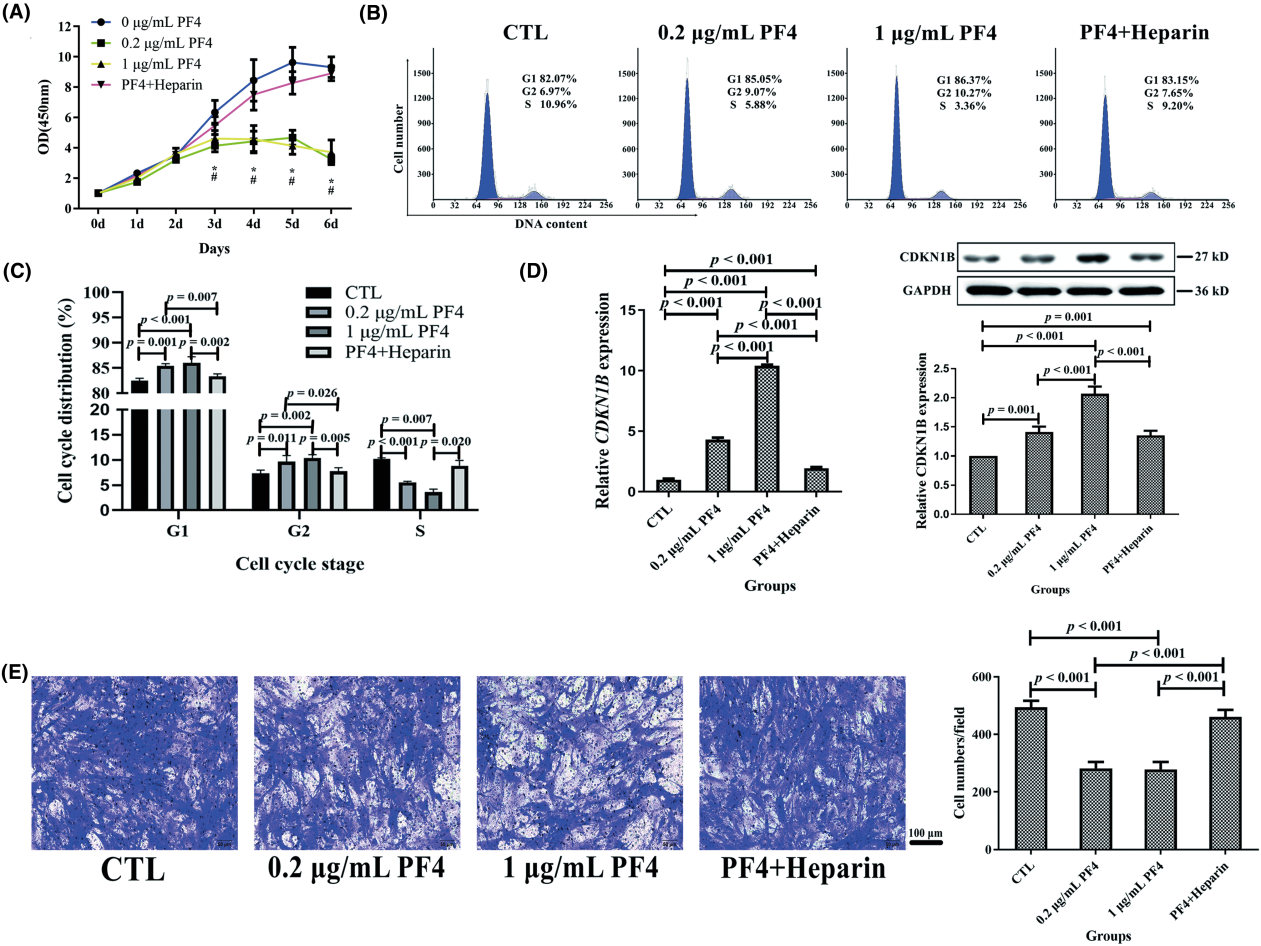
PF4 suppressed the proliferation and migration of BMMSCs in vitro. (A) The cellular proliferation rate of BMMSCs was evaluated using a CCK8 assay in four groups. (B) Representative FCM images of the cell cycle of BMMSCs in the four groups. (C) The cell‐cycle distribution on BMMSCs was detected using FCM in the four groups. (D) The relative gene and protein expression levels on CDKN1B related to the cell cycle were assessed using qPCR and WB. (E) The BMMSC migration ability was analyzed using transwell assay (stained with a crystal violet solution). Scale bar = 100 μm. *N* = 6 samples per group. Data are expressed as the mean ± standard deviation (SD) of experiments. BMMSCs, bone marrow mesenchymal stem cells; CDKN1B, cyclin‐dependent kinase inhibitor 1B; FCM, flow cytometry; GAPDH, glyceraldehyde‐3‐phosphate dehydrogenase; PF4, platelet factor 4; qPCR, quantitative real‐time polymerase chain reaction; WB, Western blotting; ^#^ *p* < 0.05 compared with 0 μg/mL PF4; *p < 0.05 compared with PF4 + heparin.

To explore the effects of PF4 on BMMSCs, the detection of cell proliferation or migration of BMMSCs was initially performed. The CCK8 assay indicated that the cell proliferation rate of the BMMSCs in the 0.2‐ and 1‐μg/mL PF4 groups was lower than that in the 0‐μg/mL PF4 and PF4 + heparin groups from Days 3 to 6 (Figure 1A). Furthermore, the cell cycle detected using FCM showed that the BMMSCs in the 0.2‐ and 1‐μg/mL PF4 groups had a markedly decreased proportion of cells in S phase compared to those in the CTL group. In addition, the proportion of cells in the S phase in the 1‐μg/mL PF4 group exhibited a significant decline compared to that in the PF4 + heparin group. Therefore, the cell counts in the G_0_/G_1_ and/or G_2_/M phase of the 0.2‐ and 1‐μg/mL PF4 groups dramatically increased compared with those in the CTL and PF4 + heparin groups (Figure 1B,C). Moreover, the relative expression of *CDKN1B* in the 0.2‐ and 1‐μg/mL PF4 groups was higher than that in the CTL and PF4 + heparin groups. Besides, the 1‐μg/mL PF4 group was associated with a dramatic increase in the relative expression of *CDKN1B* compared to the 0.2‐μg/mL PF4 group. Furthermore, the PF4 + heparin group exhibited a significant increase in the relative expression of *CDKN1B* compared to the CTL group. Moreover, the protein expression levels of CDKN1B were similar to the mRNA expression levels (Figure 1D).

In addition, a transwell assay demonstrated that the cell migration ability of BMMSCs in the 0.2‐ and 1‐μg/mL PF4 groups was lower than that of BMMSCs in the CTL and PF4 + heparin groups (Figure 1E).

### 3.2 PF4 attenuates the osteogenic differentiation ability of BMMSCs

**FIGURE 2:**
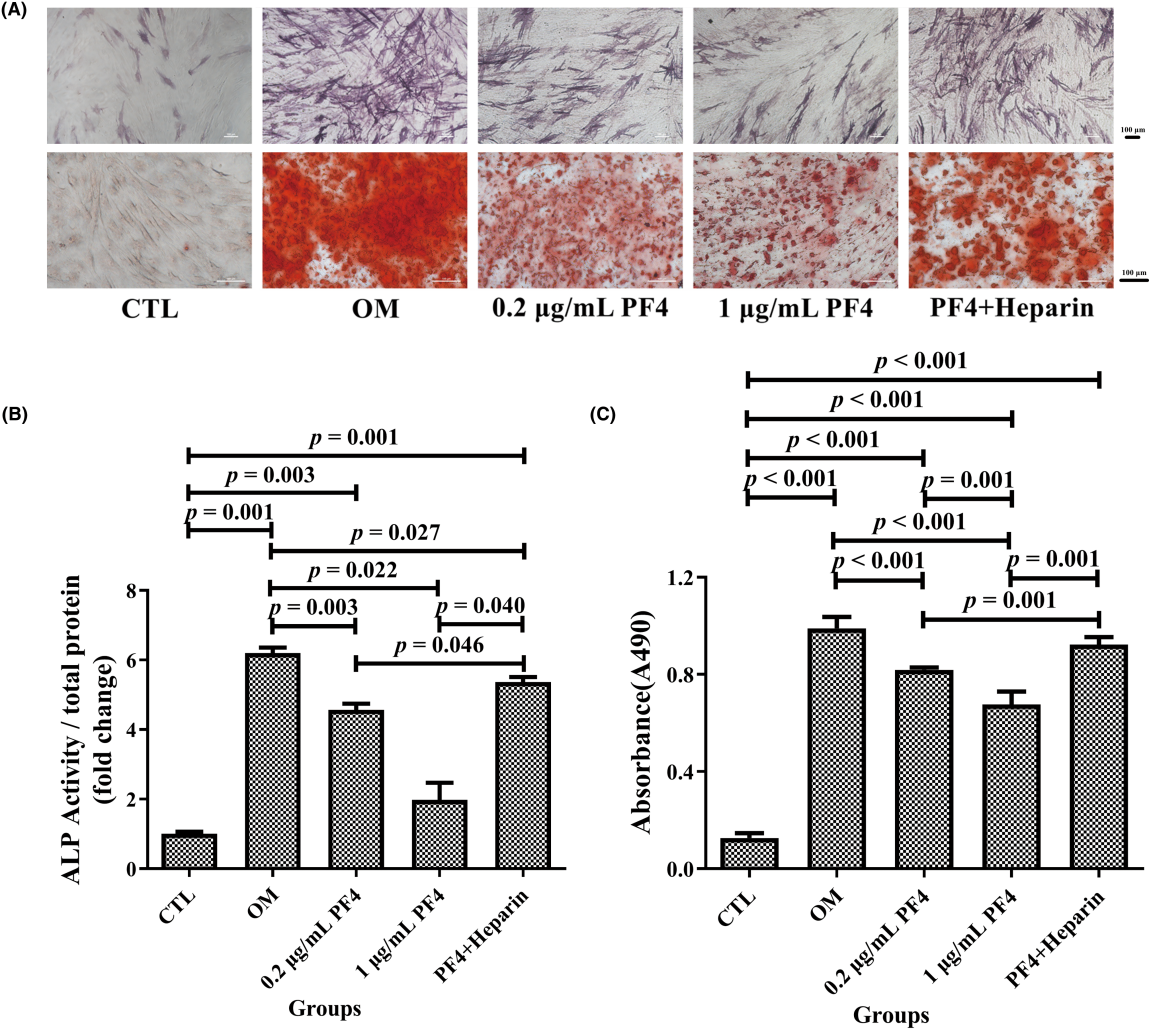
PF4 attenuated the osteogenic differentiation ability of BMMSCs in vitro. (A) Representative image of ALP and ARS staining of BMMSCs in each of the five groups. Scale bar = 100 μm. (B) ALP quantitative analysis and (C) ARS semiquantitative analysis of BMMSCs were evaluated in each of the five groups. *N* = 6 samples per group. Data are expressed as the mean ± standard deviation (SD) of experiments. ALP, alkaline phosphatase; ARS, Alizarin Red S; BMMSCs, bone marrow mesenchymal stem cells; OM, osteogenic medium; PF4, platelet factor 4.

For ALP staining and quantification, the 0.2‐ and 1‐μg/mL PF4 groups exhibited remarkably decreased ALP activity compared with the OM and PF4 + heparin groups. Furthermore, the PF4 + heparin group exhibited a significant increase in ALP activity compared with that of the CTL group and markedly decreased ALP activity compared to that of the OM group. Moreover, the ALP activity of the OM and 0.2‐μg/mL PF4 groups was higher than that of the CTL group (Figure 2A,B).

For ARS staining and quantification, the absorbance of the ARS solution dissolved in calcium nodules in the 0.2‐ and 1‐μg/mL PF4 groups was lower than that in the OM and PF4 + heparin groups and higher than that in the CTL group, which was consistent with the ALP quantification results. Moreover, the 1‐μg/mL PF4 group exhibited a remarkable decrease in the absorbance of the ARS solution compared with that of the 0.2‐μg/mL PF4 group. Moreover, the OM and PF4 + heparin groups exhibited a significant increase in the absorbance of the ARS solution compared to that of the CTL group (Figure 2A,C).

### 3.3 Trends of PF4 concentration in BM and serum post OVX

**FIGURE 3:**
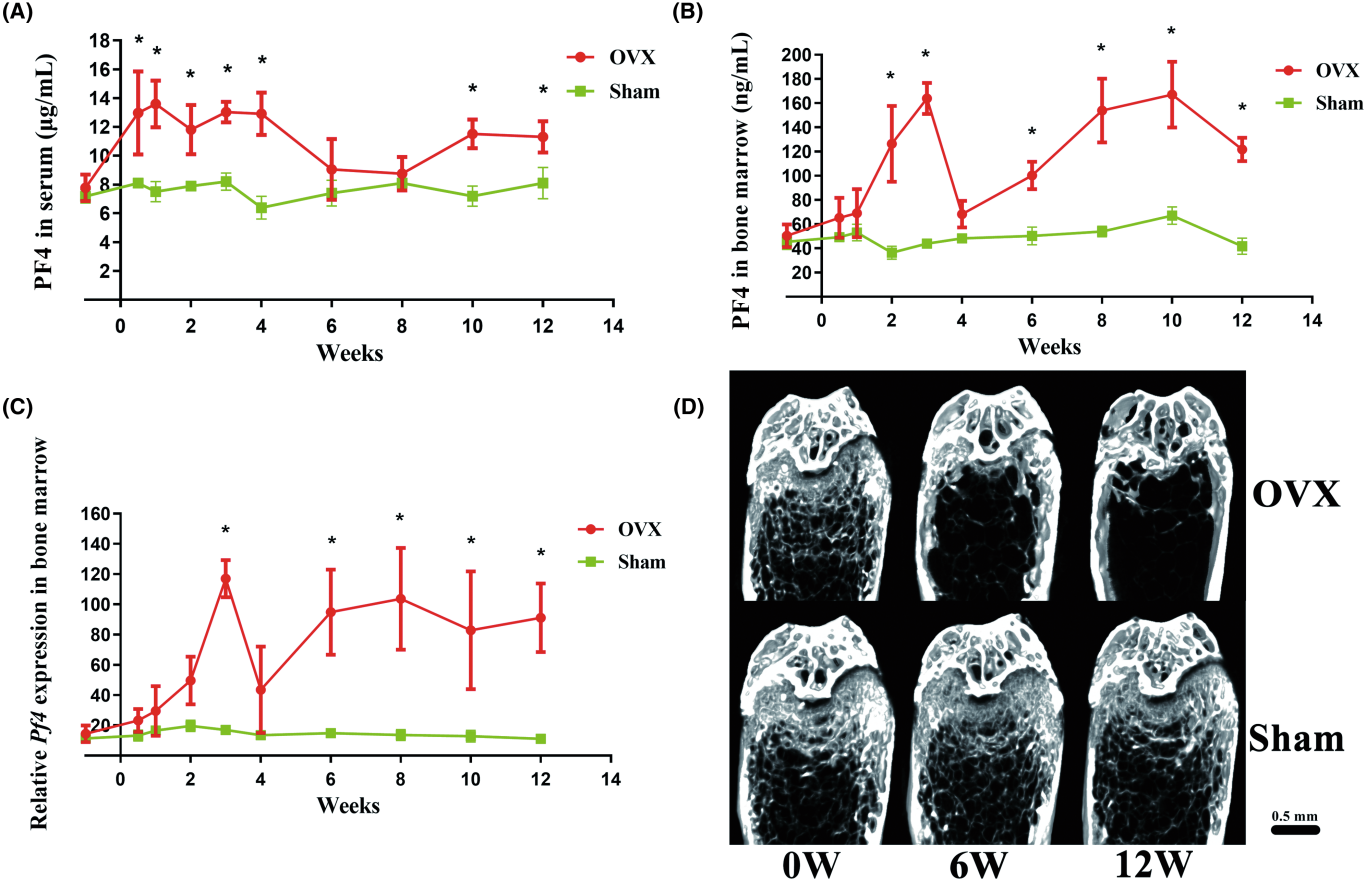
The levels of PF4 in the serum and BM generally increased in OVX mice. The variation tendency of PF4 concentrations in the (A) serum and (B) BM in OVX and sham mice (the samples were obtained every 2 weeks until 12 weeks, and the baseline was obtained 1 week before OVX). In general, the levels of PF4 in serum and BM increased in OVX mice compared with those of the sham group. (C) The variation tendency of relative *Pf4* expression in the BM of OVX and sham mice (the samples were obtained every 2 weeks until 12 weeks, and the baseline was obtained 1 week before OVX). The variation tendency of the relative expression of *Pf4* in the BM was analogous to the PF4 concentration in the BM and exhibited a more robust change. (D) Representative images of the femurs on coronal planes reconstructed using microCT in OVX and sham mice at 0, 6, and 12 weeks after OVX. The femurs of OVX mice exhibited severe bone loss at 6 and 12 weeks compared with those of sham mice. Scale bar = 0.5 mm. *N* = 6 mice per group. Data are expressed as the mean ± standard deviation (SD). *p < 0.05 compared with the sham group. BM, bone marrow; OVX, ovariectomy; PF4/*Pf4*, platelet factor 4.

To explore the relationship between the levels of PF4 and OVX‐triggered bone destruction, the variation tendency of the PF4 levels in the serum and BM was investigated in OVX and sham mice. In general, the level of PF4 was increased in OVX mice compared with that of the sham group, whereas the bone mass and microarchitecture were deteriorated due to OVX. In detail, the concentrations of serum PF4 demonstrated a significant increase after OVX, interrupted only by a temporary through/dip at ~6–8 weeks (Figure 3A). Consistent with serum PF4, the concentrations of PF4 in the BM also had a significant increase from 2 weeks, interrupted only by a temporary through/dip at ~4 weeks (Figure 3B). Moreover, the variation tendency of the relative expression of *Pf4* in the BM was analogous to the PF4 concentration in the BM and exhibited a more robust change (Figure 3C). As expected, the femurs of OVX mice exhibited severe bone loss at 6 and 12 weeks compared with those of sham mice (Figure 3D; Figure S1).

### 3.4 Supplementation with PF4 triggers bone destruction

**FIGURE 4:**
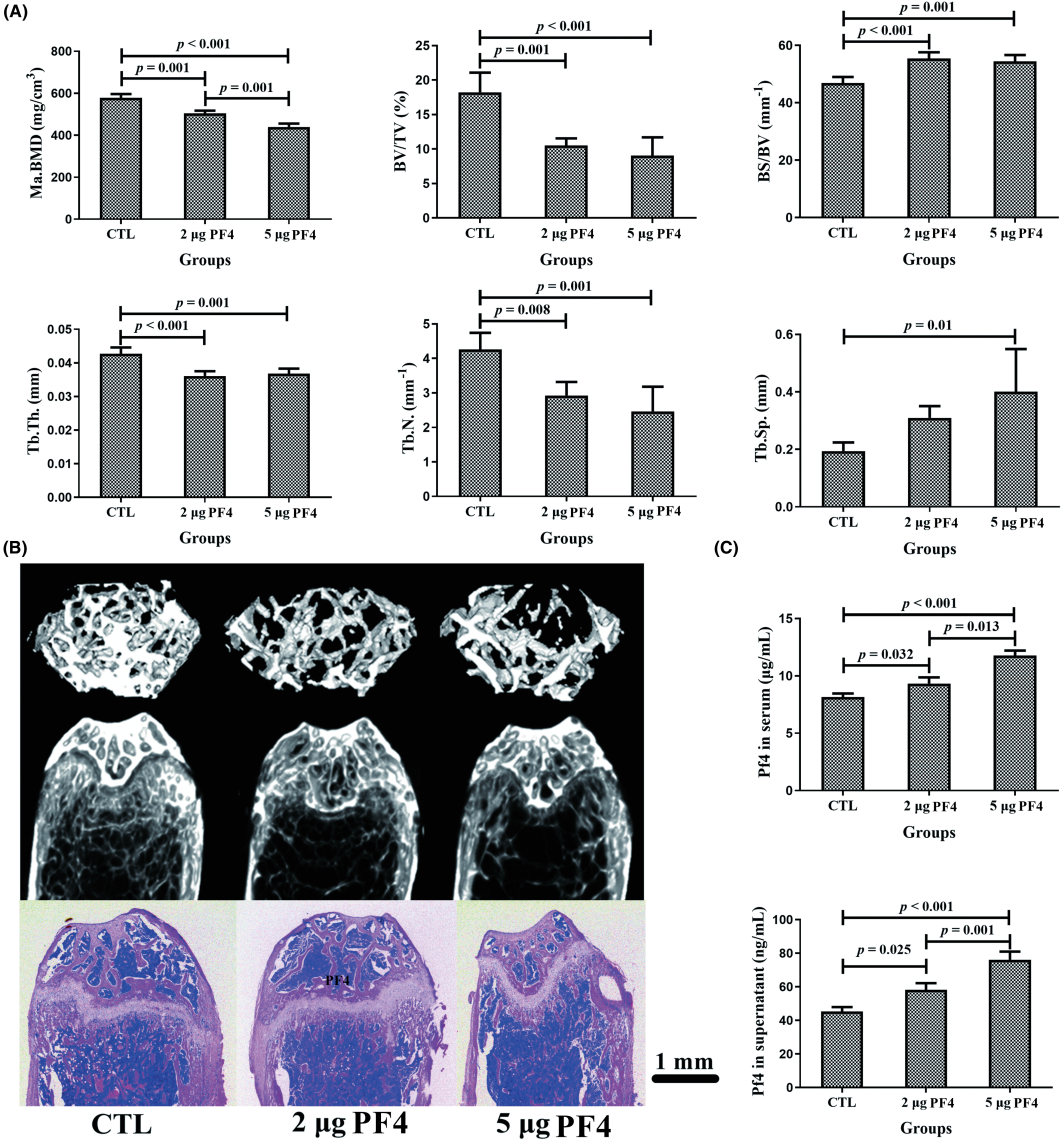
PF4 supplementation triggered bone loss in the mice at 8 weeks. (A) BMD and bone histomorphometry of the femurs in mice at 8 weeks after an injection of different doses of PF4 in three groups. In short, the BMD and bone microarchitecture of the femur decreased with increasing doses of PF4 injection. (B) Representative images of femurs reconstructed using microCT (horizontal and coronal planes) and slicing stained with H&E (coronal plane) at 8 weeks after PF4 injection in the three groups. Scale bar = 1 mm. (C) The level of PF4 in the serum and bone marrow (BM) at 8 weeks after PF4 injection in the three groups. *N* = 10 mice per group. Data are expressed as the mean ± SD (standard deviation). BS/BV, bone surface area/bone volume; BV/TV, bone volume/total volume; H&E, hematoxylin and eosin; Ma. BMD, bone mineral density of BM; PF4, platelet factor 4; Tb. N, trabecular number; Tb. Sp, trabecular separation; Tb. Th, trabecular thickness.

To further investigate the effects on PF4‐triggered bone destruction, BMD and bone microarchitecture were evaluated. In short, the BMD and bone microarchitecture of the femur decreased with increasing doses of mPF4 injection. In particular, the 2‐ and 5‐μg PF4 groups exhibited a significant decrease in Ma. BMD, bone volume/total volume (BV/TV), trabecular thickness (Tb. Th), and trabecular number (Tb. N), as well as a substantial decrease in the bone surface area/bone volume (BS/BV) compared with those in the CTL group. Moreover, the 5‐μg PF4 group exhibited a lower Ma. BMD compared to that in the 2‐μg PF4 group, as well as a higher trabecular separation (Tb. Sp) than in the CTL group (Figure 4A,B). As expected, the levels of PF4 in the serum and BM in the 2‐ and 5‐μg PF4 groups significantly increased compared to those in the CTL group. Additionally, the concentrations of PF4 in the serum and BM in the 5‐μg PF4 group exhibited significant increases compared to those in the 2‐μg PF4 group (Figure 4C).

**FIGURE 5:**
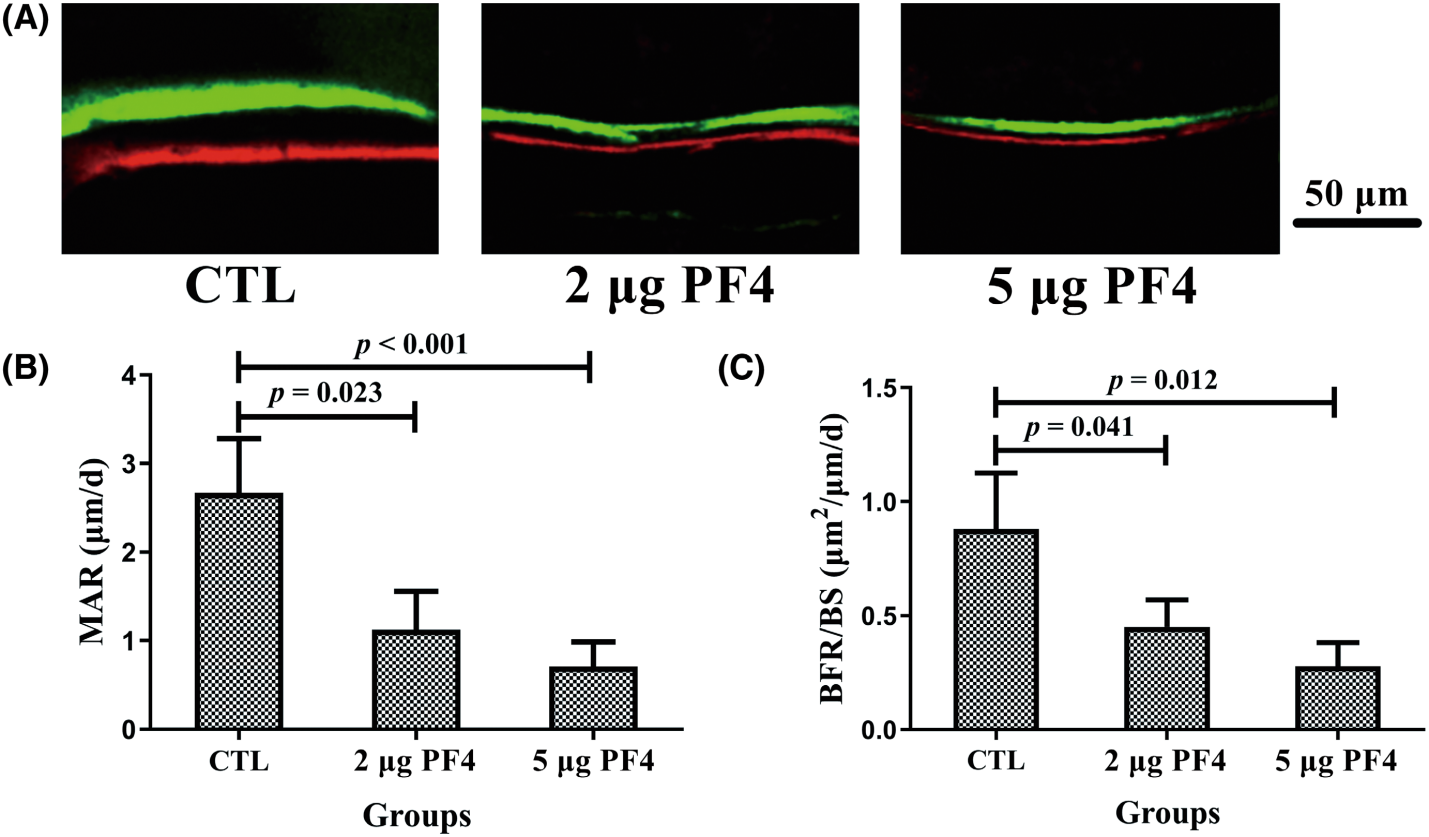
Supplementation with PF4 decreased the BFR in mice at 8 weeks. (A) Representative fluorescence images from undecalcified bone slicing of the femur after double labeling with calcein (green fluorescence band) and alizarin‐3‐methyliminodiacetic acid (red fluorescence band). The green and red fluorescence bands represent the new bone formation area on the 10th and 3rd days prior to euthanasia, respectively, and the interval of two fluorescence bands represents the BFR between 7 days. Scale bar = 50 μm. The increased area and interval of two fluorescence bands represented better bone formation ability. (B) MAR and (C) BFR/BS were evaluated in fluorescence images from undecalcified bone slicing of the femur using Bioquant software. *N* = 10 mice per group. Data are expressed as the mean ± standard deviation (SD). BFR/BS, bone formation rate/bone surface; MAR, mineral apposition rate; PF4, platelet factor 4.

In addition, there was a remarkable decline in the mineral apposition rate and bone formation rate/bone surface (BFR/BS) of the 2‐ and 5‐μg PF4 groups compared to that of the CTL group (Figure 5).

### 3.5 PF4 inhibits the ITGA5‐FAK‐ERK pathway to reduce the osteogenic differentiation of BMMSCs

**FIGURE 6:**
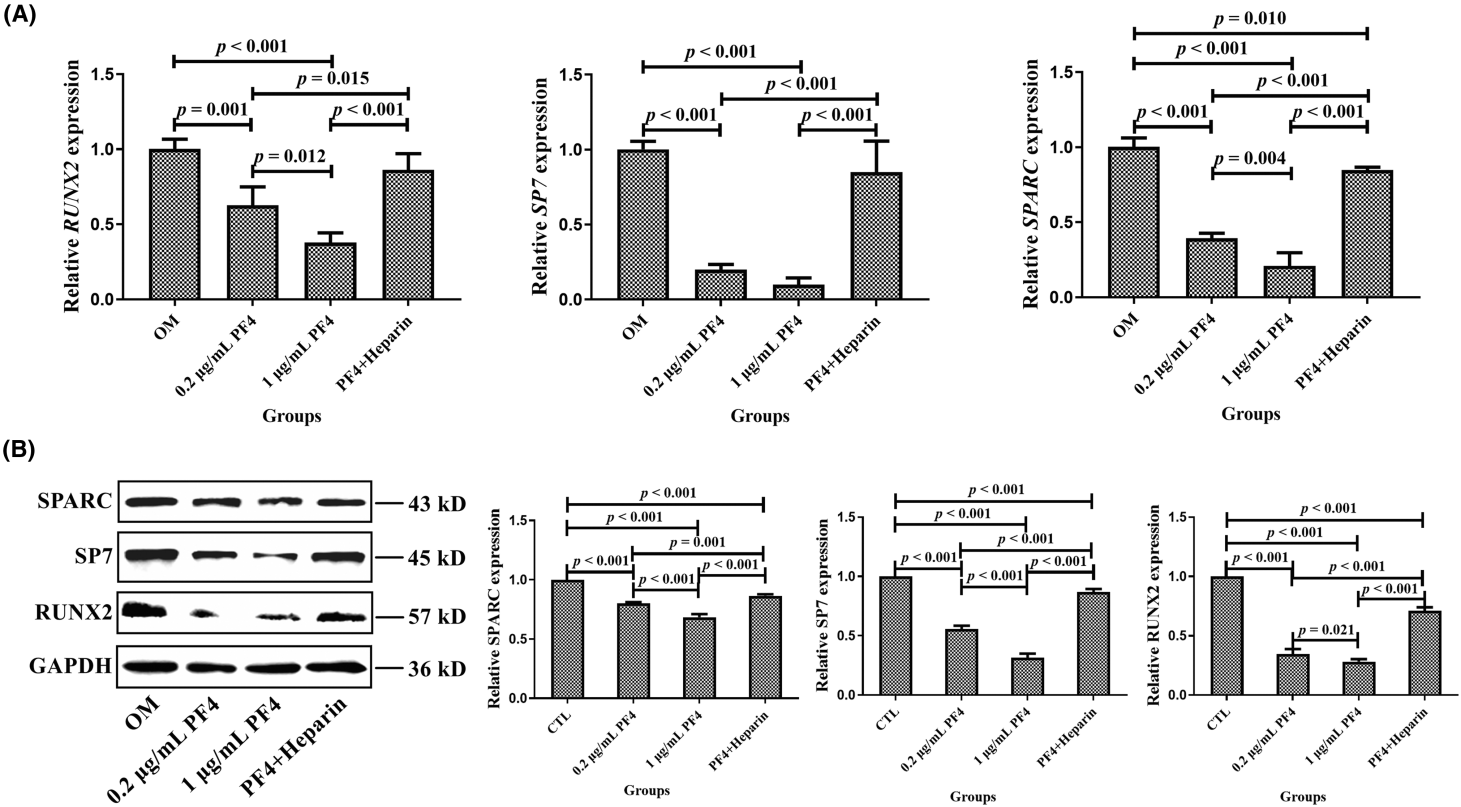
PF4 downregulated osteogenic gene and protein expression. (A) The relative gene expression levels related to osteogenesis were assessed using qPCR in the OM, 0.2‐μg/mL PF4, 1‐μg/mL PF4, and PF4 + heparin groups. (B) The relative protein expression levels related to osteogenesis were assessed using WB in the OM, 0.2‐μg/mL PF4, 1‐μg/mL PF4, and PF4 + heparin groups. *N* = 3–6 samples per group. Data are expressed as the mean ± standard deviation (SD) of the experiments. BMMSCs, bone marrow mesenchymal stem cells; GAPDH, glyceraldehyde‐3‐phosphate dehydrogenase; OM: osteogenic medium; PF4, platelet factor 4; qPCR, quantitative real‐time polymerase chain reaction; RUNX2, RUNX family transcription factor 2; SP7, Sp7 transcription factor; SPARC, secreted protein acidic and cysteine rich; WB, Western blotting.

Next, to investigate the underlying mechanisms of PF4‐mediated regulation of osteogenic differentiation of BMMSCs, some key regulators of osteogenesis were screened using qPCR. In particular, the 0.2‐ and 1‐μg/mL PF4 groups exhibited a significant decrease in the relative expression of *RUNX2*, *SP7*, and *SPARC* compared with that in the OM and PF4 + heparin groups. Moreover, the relative expression of the three aforementioned osteogenic genes (except for *SP7*) in the 1‐μg/mL PF4 group was lower than that in the 0.2‐μg/mL PF4 group (Figure 6A). Moreover, the protein expression levels of three osteogenic markers were similar to the mRNA expression levels of the aforementioned genes (Figure 6B).

**FIGURE 7:**
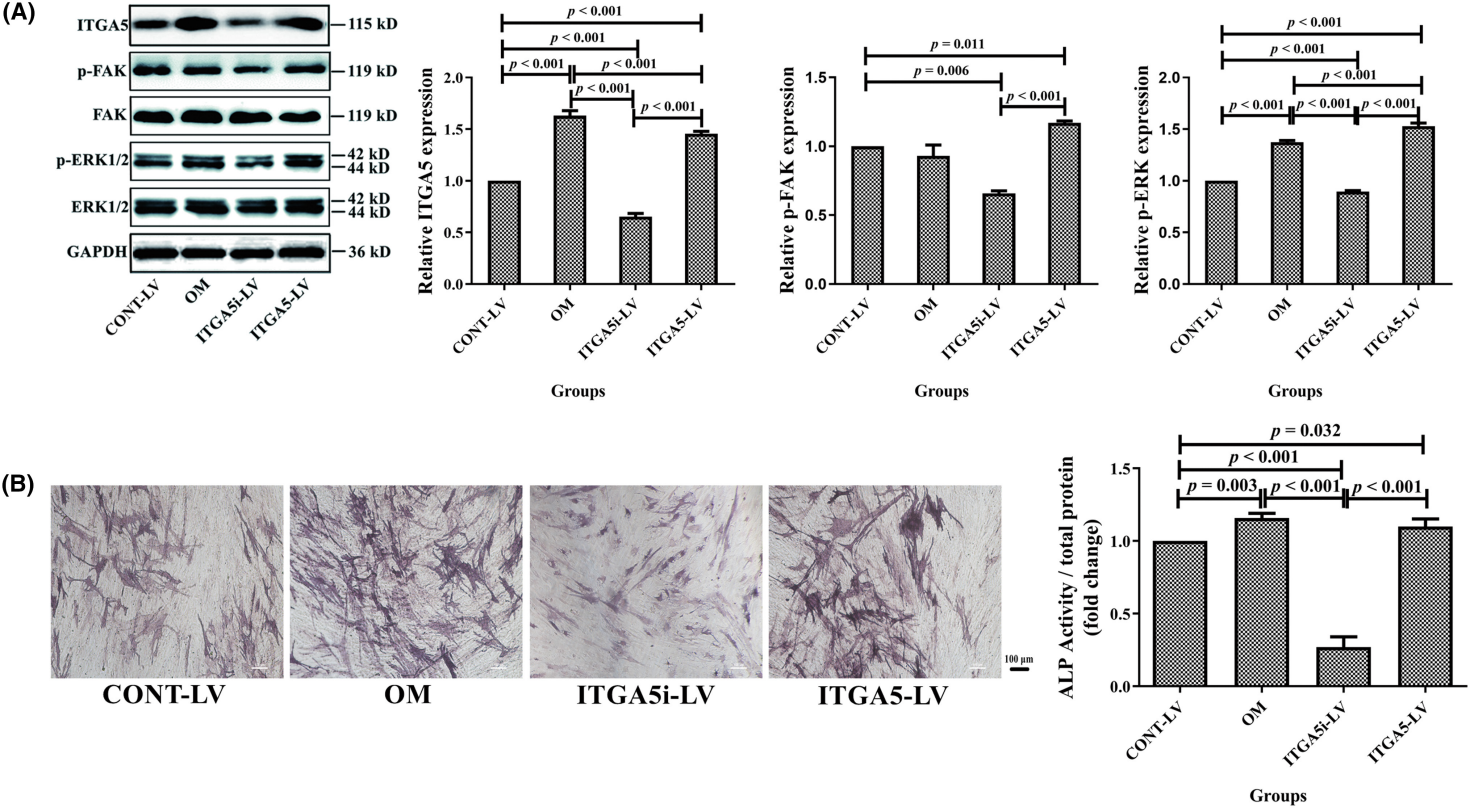
PF4 decreased the ALP (alkaline phosphatase) activity of BMMSCs by inhibiting the ITGA5‐FAK‐ERK pathway. (A) The protein expression levels of ITGA5, p‐FAK, FAK, p‐ERK1/2, and ERK1/2 were evaluated among the CONT‐LV, OM, ITGA5i‐LV, and ITGA5‐LV groups. (B) Representative images of staining and quantitative analysis of ALP for BMMSCs among the CONT‐LV, OM, ITGA5i‐LV, and ITGA5‐LV groups. Scale bar = 100 μm. *N* = 3–6 samples per group. Data are expressed as the mean ± standard deviation (SD) of the experiments. BMMSCs, bone marrow mesenchymal stem cells; CONT‐LV, control lentivirus; GAPDH, glyceraldehyde‐3‐phosphate dehydrogenase; ITGA5, integrin α5; ITGA5‐LV, ITGA5 overexpression lentivirus; ITGA5i‐LV, ITGA5 interfering lentivirus; OM, osteogenic medium; PF4, platelet factor 4; p‐ERK, phosphorylated extracellular regulated protein kinases; p‐FAK, phosphorylated focal adhesion kinase.

Furthermore, the protein expression level of ITGA5 decreased in the ITGA5 interfering lentivirus group and increased in the ITGA5 overexpression lentivirus group compared with the control lentivirus group, which was similar to the protein expression levels of p‐FAK, p‐ERK1/2, and ALP (Figure 7).

## 4 DISCUSSION

This study revealed that PF4 widely decreased cell proliferation, cell cycle, cell migration, and osteogenic differentiation of BMMSCs. Therefore, research into the molecular mechanisms of osteogenesis validated the downregulation of several key regulators of osteogenesis, and the ITGA5‐FAK‐ERK pathway was inhibited due to PF4 supplementation. Further investigation demonstrated that the level of PF4 in the serum and BM generally increased, whereas the bone mass and microarchitecture deteriorated in mice as a result of OVX. Furthermore, in vivo supplementation with mPF4 triggered bone loss in mice due to the suppression of bone formation.

In the present study, the proliferation and cell cycle of BMMSCs were suppressed by PF4. Previous studies also validated the ability of PF4 to maintain the cell quiescence of HSCs to regulate the survival and self‐renewal of HSCs,^21^, ^38^ which was consistent with our results. It is important to note that it was moderate and reversible to suppress the cell proliferation and cell cycle of BMMSCs to promote cell survival and self‐renewal rather than cell death. Therefore, based on the in vivo and in vitro results, we speculated that at first, BMMSCs rapidly underwent proliferation and osteogenic differentiation to avoid quick bone loss after OVX. However, PF4 maintained the BMMSC population and pluripotency to prevent excessive BMMSC exhaustion. Finally, the balance was disrupted, and OVX‐induced bone loss occurred because of consistently high levels of PF4, which may indicate that it is more important to maintain the BMMSC population and pluripotency for the entire body than to rescue bone loss only. The BMMSC regulation requires further investigation.

Our previous study revealed that aspirin prompted in vitro cell migration and in vivo cell homing of adipose‐derived stromal cells^39^; however, the associated mechanisms remain unknown. Interestingly, this study revealed that cell migration of BMMSCs was suppressed by PF4 in a dose‐dependent manner. Of note, recent research has supported the hypothesis that the systemic administration of aspirin induced a significant decrease in the level of PF4 in a rat model of periodontal disease.^40^, ^41^ Overall, these studies suggested that aspirin promoted the cellular migration of stem cells due to the inhibition of PF4 levels.

In addition, the results demonstrated that PF4 attenuated the osteogenic differentiation of BMMSCs; however, Chen et al. showed that ALP staining of the PF4 group was weaker than that of the CTL group, which was similar to the results of our study.^42^ Further research revealed that PF4 downregulated five key regulators of osteogenesis and inhibited the ITGA5‐FAK‐ERK pathway to decrease the osteogenic differentiation of BMMSCs. Aidoudi et al. deemed that PF4 could interact with the integrins αvβ3/αvβ5/α5β1 to suppress endothelial cell adhesion and migration and further contribute to its antiangiogenic effect.^24^ Moreover, the study by Kulkarni and Jackson revealed that platelet factor XIII limited platelet aggregate formation and thrombus growth by regulating the integrin αIIbβ3.^22^ In addition, Lishko et al. demonstrated that leukocyte integrin Mac‐1 functions as a functional receptor for PF4.^23^ In this study, we also found that PF4 could regulate ITGA5 to attenuate osteogenic differentiation, which is consistent with the findings of the aforementioned studies. It is important to note that recent research has validated that the integrin‐FAK‐ERK pathway plays a vital role in bone metabolism. For example, Viale‐Bouroncle et al. found that laminin regulated the osteogenic differentiation of dental follicle cells through the integrin α2β1‐FAK‐ERK pathway.^43^ Moreover, Chandran et al. showed that a biocomposite scaffold containing the phytomolecule diosmin could promote osteoblast differentiation and bone regeneration in mouse BMMSCs through the integrin‐FAK‐ERK pathway.^44^ In addition, Chen et al. reported that osteopontin increased the migration of chondrosarcoma cells through the integrin αvβ3, FAK, and ERK pathways.^45^ Furthermore, another study found that osteopontin promoted MSC migration via the integrin β1, FAK, and ERK pathways.^46^

In this study, we first found that the level of PF4 generally increased in OVX mice compared with sham mice. Of note, the levels of PF4 fluctuated twice prior to euthanasia rather than consistently increasing. In particular, the concentration of PF4 in the BM increased rapidly after OVX and peaked in the third week. However, the PF4 level declined quickly in the fourth week and slowly increased again between the 6th and 10th weeks. Surprisingly, the level of PF4 began to decline at 12 weeks. As expected, the variation tendency of the PF4 level in the serum was similar to that in BM. Considering that PF4 is stored in platelet α‐granules and can be quickly released into the serum or BM,^14^ we speculated that the first peak of PF4 was from platelets. After PF4 in platelets became exhausted, PF4 was synthesized by megakaryocytes and gradually released into the serum or BM.^21^ Therefore, the PF4 level increased again and was maintained at a higher level in OVX mice than in the sham group. Furthermore, we found that supplementation with PF4 triggered decreased BMD and deterioration of bone microarchitecture, which means that PF4 indeed played a key role in bone loss. Next, the correlation between PF4 and bone loss in postmenopausal women will be explored in the future.

There are some limitations of this study. In vivo mechanisms of increased PF4‐triggered bone loss should be explored to confirm the cause‐and‐effect relationship between PF4 and bone. In addition, treatments for bone loss triggered by PF4, such as PF4 neutralization antibodies, should be further investigated.

## 5 CONCLUSIONS

PF4 may be related to OVX‐induced bone loss triggered by the suppression of bone formation in vivo. Furthermore, PF4 alleviated BMMSC osteogenic differentiation by inhibiting the ITGA5‐FAK‐ERK pathway.

## AUTHOR CONTRIBUTIONS

Hao Liu conceived and supervised the study, designed the experiments, and analyzed the data; Wei Li, Ranli Gu, Lijun Zeng, and Qiwei Zhang performed the experiments and analyzed the data. Wei Li and Ranli Gu wrote the manuscript; Qiwei Zhang and Wei Li revised the manuscript.

## FUNDING INFORMATION

This work was supported by grants from the National Natural Science Foundation of China (81700935), the Beijing Natural Science Foundation (L222145), Clinical Medicine Plus X—Young Scholars Project, Peking University, the Fundamental Research Funds for the Central Universities (PKU2023LCXQ017), and CAMS Innovation Fund for Medical Sciences (2019‐I2M‐5‐038).

## CONFLICT OF INTEREST STATEMENT

The authors have no conflicts of interest to declare.

## ETHICS STATEMENT

All animal experiments were approved by the Animal Care and Use Committee of Peking University Health Science Center (approval number: LA2019019; Beijing, China).

## Supporting information


Figure S1.

Table S1.
