# Supplementary material for: Platelet factor 4 induces bone loss by inhibiting the integrin α5‐FAK‐ERK pathway
Source: Animal Model Exp Med. 2023 Aug 11;6(6):573–84. doi: 10.1002/ame2.12342 (PMC10757219; doi:10.1002/ame2.12342)
Supplement: Supplementary file 1 — Figure S1. [file AME2-6-573-s001.docx]

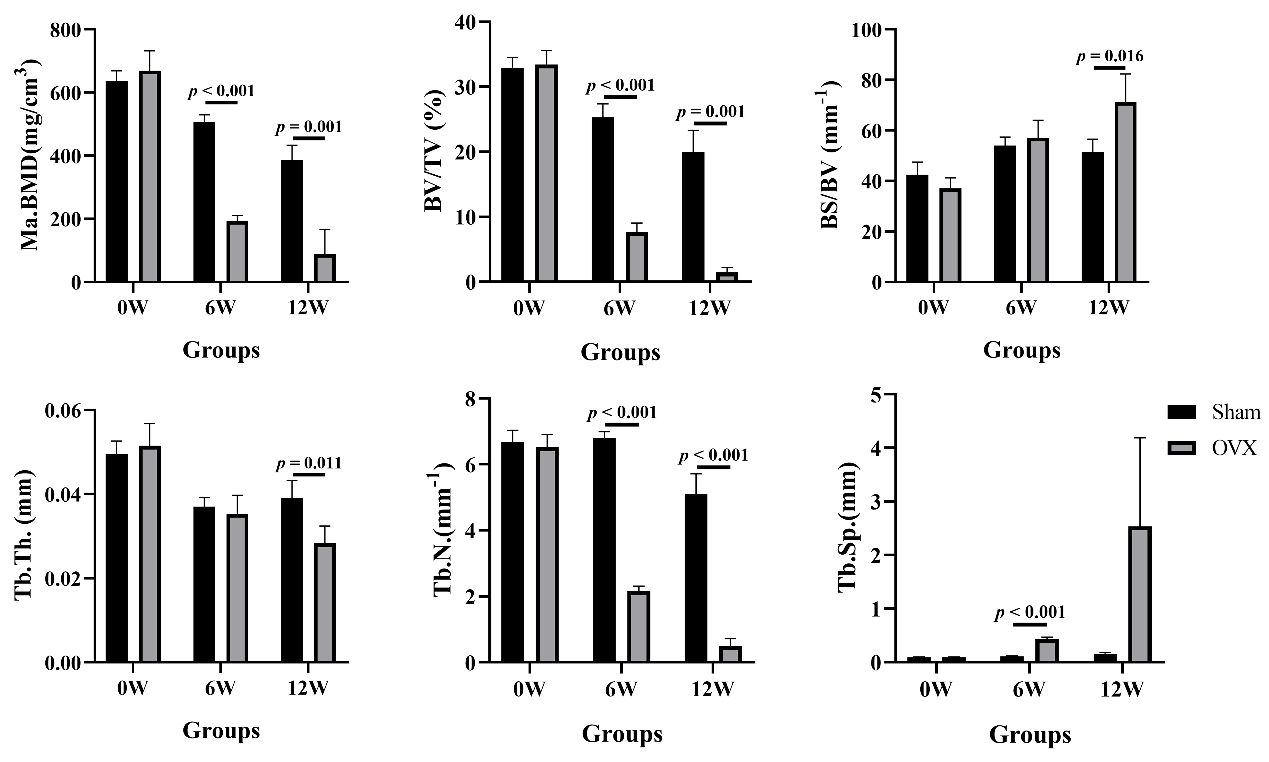


**FIGURE S1** Comparison on BMD and bone histomorphometry of the femurs between Sham and OVX mice at 0, 6, and 12 weeks. Briefly, femur of OVX mice exhibited a severe bone loss at 6 and 12 weeks compared with those of Sham mice (n = 6).
