# Supplementary material for: Platelet factor 4 induces bone loss by inhibiting the integrin α5‐FAK‐ERK pathway
Source: Animal Model Exp Med. 2023 Aug 11;6(6):573–84. doi: 10.1002/ame2.12342 (PMC10757219; doi:10.1002/ame2.12342)
Supplement: Supplementary file 2 — Table S1. [file AME2-6-573-s002.docx]

**Tables**

**TABLE 1.** Sequences of the primers used for qPCR.

|  | **Forward primer (5’ to 3’)** | **Reverse primer (5’ to 3’)** |
| --- | --- | --- |
| *GAPDH* | CGGACCAATACGACCAAATCCG | AGCCACATCGCTCAGACACC |
| *PF4* | TGGAGGTGATCAAGGCGGGAC | GGCAGCTTCTACCTAACTCTCCA |
| *RUNX2* | ACTACCAGCCACCGAGACCA | TGGCAGGTACGTGTGGTAGT |
| *SP7* | CCTCCTCAGCTCACCTTCTC | GTTGGGAGCCCAAATAGAAA |
| *SPP1* | ATGATGGCCGAGGTGATAGT | ACCATTCAACTCCTCGCTTT |
| *SPARC* | AGTGCACCCTGGAGGGCACC | TGCTTGATGCCGAAGCAGCC |
| *COL1A1* | GAGGGCCAAGACGAAGACATC | CAGATCACGTCATCGCACAAC |
| *ITGA5* | TTCGCCTCTGGGAGGTTTAG | TCCGCGTCTAAGTTGAAGCC |
| *CCND1* | GGATGCTGGAGGTCTGCGA | AGAGGCCACGAACATGCAAG |
| *CDK4* | AGTTCGTGAGGTGGCTTTA | GGGTGCCTTGTCCAGATA |
| *CDKN1B* | ATGTCAAACGTGCGAGTGTC | TCTGTAGTAGAACTCGGGCAA |
| *Gapdh* | TGCACCACCAACTGCTTAGC | GGCATGGACTGTGGTCATGAG |
| *Pf4* | TGGAGGTGATCAAGGCTGGAC | GGCAGCTCTTAGCTAACTCTCCA |

*GAPDH/Gapdh,* glyceraldehyde-3-phosphate dehydrogenase; *PF4/Pf4,* platelet factor 4; *RUNX2,* RUNX family transcription factor 2; *SP7,* Sp7 transcription factor; *SPP1,* secreted phosphoprotein 1; *SPARC,* secreted protein acidic and cysteine rich; *COL1A1,* collagen type I alpha 1 chain; *ITGA5,* integrin subunit alpha 5; *CCND1*, cyclin D1; CDK4, cyclin-dependent kinase 4; CDKN1B, cyclin-dependent kinase inhibitor 1B*.*
